# Authors' Reply: Sprinkles as a Home Fortification Strategy to Improve the Quality of Complementary Foods

**DOI:** 10.1371/journal.pmed.0020202

**Published:** 2005-07-26

**Authors:** Stanley H Zlotkin, Claudia Schauer, Anna Christofides, Waseem Sharieff, Mélody C Tondeur, S. M. Ziauddin Hyder

**Affiliations:** **1**Hospital for Sick Children and University of TorontoToronto, OntarioCanada; **2**Hospital for Sick ChildrenToronto, OntarioCanada

We are writing in response to the letter by Stacia Nordin [1]. Independent of where a child is born in the world, the most appropriate feeding regimen is breast milk until six months of age, followed by a weaning or complementary food [2]. It is known that breast milk provides all the essential nutrients for a growing infant, except for vitamin D. It is also known that complementary foods should contribute to providing all of the essential nutrients when breast milk is no longer the sole source of nutrition after the first few months of life. However, as early as 1930, it was realized that typical complementary foods were generally poor sources of micronutrients (minerals and vitamins) and were often not sufficient to meet the micronutrient needs of growing children. For example, per 100 g, rice-based complementary foods contain about 1 mg of iron, and wheat-based complementary foods contain about 0.8 mg of iron [3]. Even a meat-based complementary food, such as commercial “toddler beef stew”, contains only 1.2 mg of iron in each 170-g jar. Since the recommended dietary allowance for iron is 11 mg/day (for ages 7–12 months) [4], clearly a rice- or wheat-based complementary food, or even a dilute meat-based stew, would not provide an adequate amount of iron for a growing infant.

Pablum, the first fortified baby food, was invented at the Hospital for Sick Children in Toronto, Canada, in the 1930s [5]. Subsequently, by the early 1960s in North America, all commercially manufactured infant cereals were fortified with iron. Today, the major source of iron in the diet of a North American child is fortified commercial infant cereals. And, indeed, the low rates of iron-deficiency anaemia in Canada and the United States are thought to be partly a result of the widespread use of commercially available iron-fortified cereals [6].

Another good example of a fortified food for young children in North America is fluid milk products, which are fortified with vitamin D in order to prevent the development of rickets. It is currently well accepted among nutritionists and pediatricians that most young children in North America depend on fortified foods to meet their micronutrient needs.

In most developing countries, access to commercially processed baby foods (fortified with iron) is very limited mainly because of their high cost and limited availability [7]. It is noteworthy that recent research has demonstrated that even if dietary diversification and modification (such as soaking, fermentation, and germination) strategies are used at the household level, they may not be sufficient to overcome the deficits in iron and other micronutrients [3]. As a result, other options need to be considered for young children living in developing and poor countries to ensure that all of their nutrient requirements are met [8]. The use of Sprinkles is one such option [9,10]. One of the greatest advantages of the Sprinkles concept is its emphasis on complementary food consumption because Sprinkles have to be mixed with food. When educating caregivers about anaemia and the use of Sprinkles, healthy weaning practices can be concurrently promoted to ensure the timely introduction of complementary foods at six months of age in addition to continued breast feeding (as recommended by the World Health Organization) [2]. This is an important benefit, as it is well known that in many developing countries poor weaning practices are common [3]. As a home fortificant, Sprinkles ensure that the food eaten contains adequate amounts of essential micronutrients. Indeed, Sprinkles are meant to improve the nutritional value of homemade baby foods, which are otherwise poor in micronutrient content. Sprinkles can enrich foods not only with iron but also with other essential micronutrients such as zinc, folic acid, and vitamins A and C. In addition, since Sprinkles can be easily mixed with any homemade semisolid foods, their use does not require any change in food practices; thus, they can be easily accepted in diverse cultural settings.

With anaemia rates as high as 80% in young children in some developing countries, current food-based strategies alone are clearly not effective. All children should have the right to eat foods that meet their nutritional needs. The use of Sprinkles is one way to help these children meet their nutrient requirements. Unfortunately, a food-based strategy alone, using locally available unfortified foods, in most circumstances, is simply inadequate and may further predispose a growing child to various micronutrient deficiencies [8].
